# *In situ* observations on the dentition and oral cavity of the Neanderthal skeleton from Altamura (Italy)

**DOI:** 10.1371/journal.pone.0241713

**Published:** 2020-12-02

**Authors:** Alessandro Riga, Marco Boggioni, Andrea Papini, Costantino Buzi, Antonio Profico, Fabio Di Vincenzo, Damiano Marchi, Jacopo Moggi-Cecchi, Giorgio Manzi

**Affiliations:** 1 Department of Biology, University of Florence, Florence, Italy; 2 Laboratory of Archaeoanthropology, SABAP-FI, Scandicci (FI), Italy; 3 School of Paleoanthropology, Perugia, Italy; 4 Department of Environmental Biology, Sapienza University of Rome, Roma, Italy; 5 Department of Archaeology, University of York, York, United Kingdom; 6 Natural History Museum, University of Florence, Florence, Italy; 7 Department of Biology, University of Pisa, Pisa, Italy; 8 Evolutionary Studies Institute and Centre for Excellence in PalaeoSciences, University of the Witwatersrand, Johannesburg, South Africa; 9 Istituto Italiano di Paleontologia Umana, Anagni, Roma, Italy; Max Planck Institute for the Science of Human History, GERMANY

## Abstract

The Neanderthal specimen from Lamalunga Cave, near Altamura (Apulia, Italy), was discovered during a speleological survey in 1993. The specimen is one of the most complete fossil hominins in Europe and its state of preservation is exceptional, although it is stuck in calcareous concretions and the bones are mostly covered by calcite depositions. Nevertheless, it is possible to carry out some observations on craniodental features that have not previously been described. In this work, we present an account of the oral cavity, made possible by the use of a videoscope, which allowed us to reach some hidden parts of the mandible and palate. This is the first detailed overview of the teeth and maxillary bones of the Neanderthal skeleton from Altamura. The dentition is almost complete. However, two teeth (upper right P3 and upper left M1) were lost *ante mortem* and four teeth (lower right I1 and P3 and lower left I1 and I2) were lost most probably *post mortem*. Dental wear is marked. The erupted M3s and the inversion of the compensating curve of Wilson in the M1s and M2s but not in the M3s suggest that the individual is fully adult, but not old. Although most of the teeth have their roots exposed for several millimeters, the periodontal bone appears to be in good condition overall, except in correspondence of the two *ante-mortem* tooth losses. X-rays of the anterior teeth show a periapical lesion, probably linked to the advanced dental wear. We also observed a weak expression of taurodontism in the posterior dentition and the presence of a retromolar space, features consistent with an attribution to the Neanderthal hypodigm; this attribution is also supported by aspects of the cranial morphology, the morphometric analysis of the scapula and preliminary mtDNA data. There is also a well-developed palatine torus, to the best of our knowledge a feature not previously described in Neanderthals.

## Introduction

The Neanderthal skeleton from Altamura (Apulia, Italy), discovered in 1993 [1], is the most complete Neanderthal ever found and possibly the most complete fossil hominin ever found before modern humans. It was discovered in a branch of Lamalunga Cave (40° 52' 18.64" N, 16° 35' 14.98" E), which is the upper part of a wider karstic complex in the Murgia plateau. This geographic region of southern Italy is rich in karstic formations such as dolines, caves and blind valleys [2].

**Fig 1:**
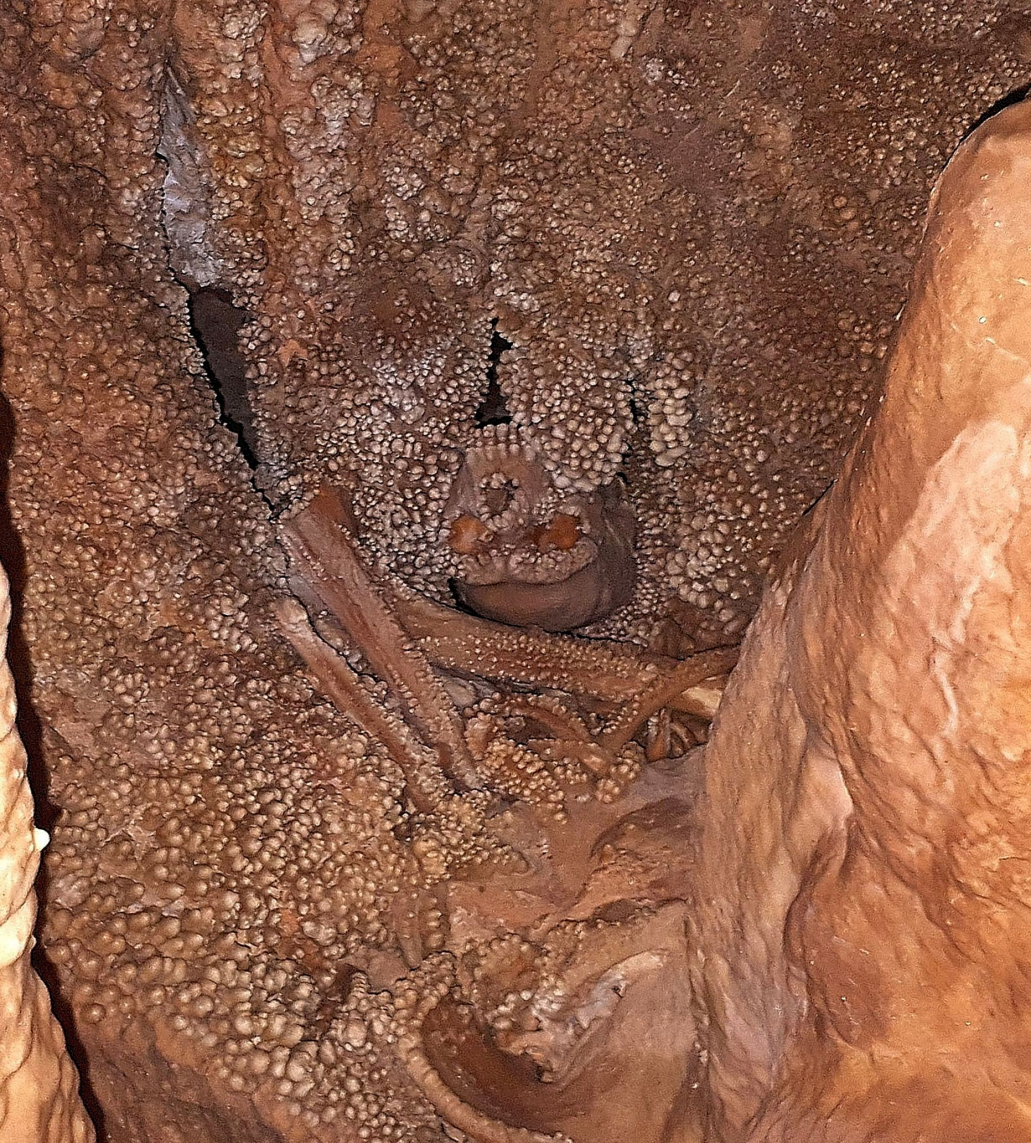
The “Apse of Man”. The Altamura specimen lies in a corner of this small chamber, mostly embedded in calcareous formations and covered by calcite layers and coralloid deposits.

The skeleton lies in a corner of a small chamber (Fig 1), called the “Apse of Man”, delimited by speleothemic formations and embedded in calcareous formations, with all the bones covered by a calcite layer and coralloid deposits [1, 3]. Faunal elements found in the cave were probably scattered by water, whereas all the bones of the hominin specimen are concentrated in a small area and it is likely that the skeleton now lies approximately where the body decomposed [4, 5].

Observations of the skeletal features suggest a combination of archaic and Neanderthal derived traits, placing the specimen in the human variability of the Late-Middle Pleistocene in Europe [6]. Between 2009 and 2015, a large part of its fragmentary right scapula was removed from the cave in three pieces [3, 7, 8]. In particular, the portion comprising the glenoid fossa, the neck and the roots of the spine and coracoid process of the scapula was extracted in 2009 and its study provided information on paleogenetics and morphology [3]. The morphometric analysis of the glenoid fossa confirmed that the specimen falls within the Neanderthal range of variation, despite some morphological peculiarities [3, 8]. At the same time, paleogenetic data confirmed the Neanderthal attribution [3] of the specimen, while U/Th dating suggested that the individual died between 130.1 ± 1.9 ka and 172 ± 15 ka [3].

Within the context of a broader project coordinated by one of us (GM) and financed by the Italian Ministry of Instruction, University and Research (MIUR; PRIN call 2015), new research activities were recently carried out in the cave. During this new stage, high-definition photographic and three-dimensional (3D) digital images were acquired by different techniques, allowing the observation of several new features of the Altamura Neanderthal despite the presence of the calcareous concretions partially covering the specimen. In this work, we present the first observations carried out *in situ* on the dentition and oral cavity.

## Materials and methods

**Fig 2:**
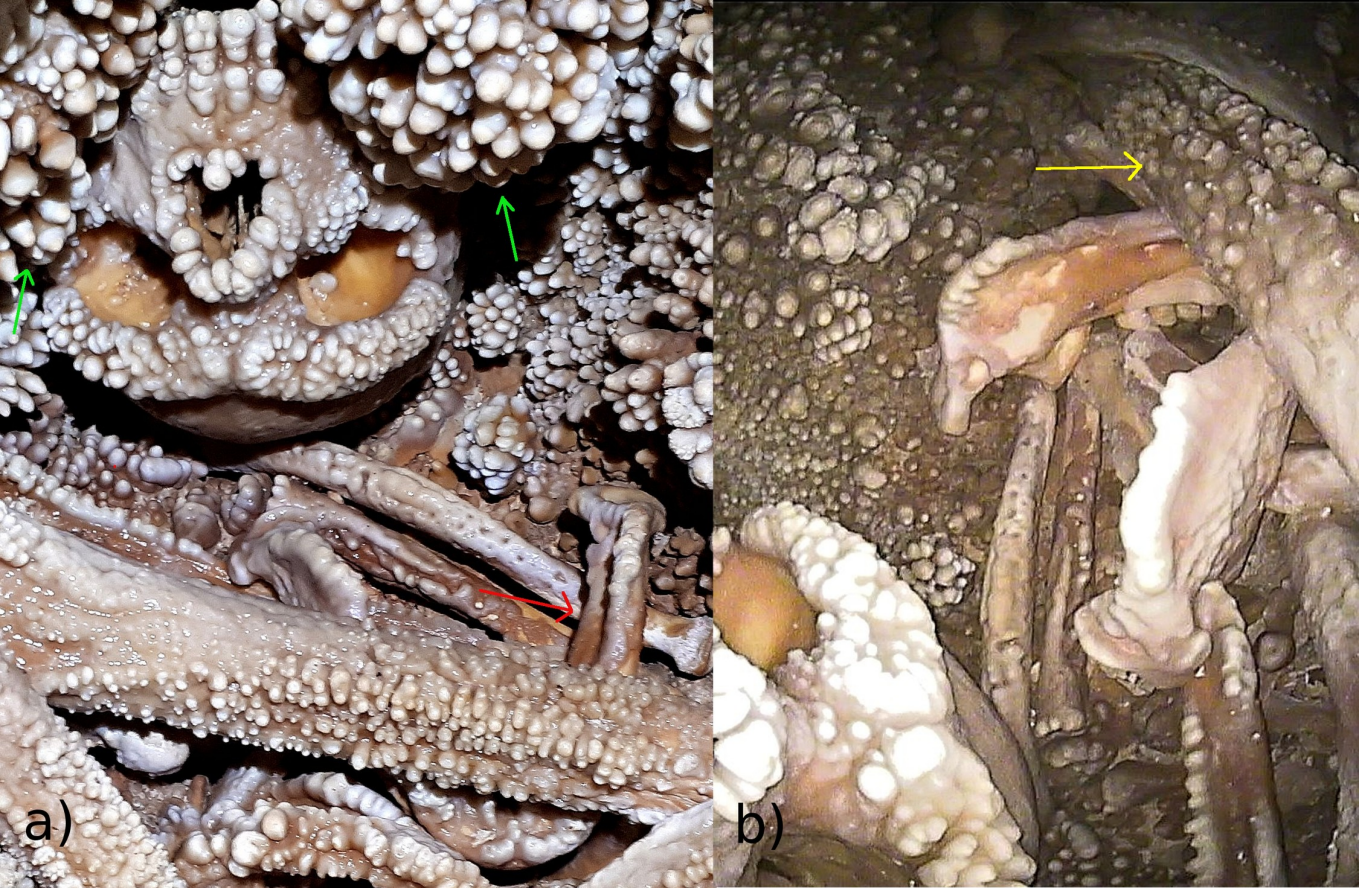
Cranium and mandible. a) the cranium lies on its vault, embedded between two large calcareous formations (green arrows), while the mandible is in front of the cranium, slightly to its right (red arrow). b) The mandible lies upside down above other bones and the right femur (blue arrow) rests on it, covering the anterior part of the body and the mandibular symphysis.

To understand what kind of observations would be possible, it was important to figure out the relative position of the cranium and mandible in the cave (Fig 2). The cranium lies on its vault, slightly tilted to the left, with the orbits facing anteriorly toward the ‘Apse’. Two large apices of karstic concretions descend from above, framing the midface on the right and the left side of the cranium, thus preventing direct observation of the entire maxillary arcade and teeth. Only the labial aspect of the incisors can be easily examined. The mandible is located in the bone assemblage in front of the cranium and slightly to its right (Fig 2). It lies upside down with the teeth facing the floor of the cave. The occlusal surfaces of the molar teeth are in partial contact with the right fibula below them. The right femur rests above the anterior part of the body of the mandible, close to the symphysis.

Overall preservation of the dentition and oral structures is good; however, all surfaces (teeth and bones) are covered with a calcite layer of variable thickness. Moreover, large “coralloid” formations (as defined in [3]) cover the tooth crowns in some areas, notably the maxillary anterior teeth, partially hiding the original morphology of the labial, incisal and lingual surfaces (Fig 2A). Because of the position of the cranium and mandible and the widespread calcite layers covering all bones and teeth, morphological observations were limited to the gross morphology of the oral structures, and any analysis had to be carried out with the help of various technical devices.

Photos of the dentition, maxilla and mandible were taken with an Olympus IPLEX NX IV9635N videoscope with a 3.5 m-long probe of 6 mm diameter and 120D/NF lens with a 120° angle; the base unit was an 8.4-inch daylight view LCD touchscreen monitor. The small dimensions and the high flexibility of the Olympus probe meant that it could be inserted through the narrow passages of the karstic flow above the cranium to provide images of the lingual side and occlusal plan of the maxillary arch and to reach the mandible below the bone assemblage. Prior to the use of the Olympus videoscope, test acquisitions were made with an Ambu aScope 3 videoscope with aView LCD monitor.

The position of the skull and mandible prevented us from using X-ray devices on all the dentition and we could only take X-ray images of the upper anterior teeth with a handheld KaVo NOMAD^™^ Pro 2 X-ray system (exposure time 1 sec, 60 kV, 2.5 mA, distance from subject 35 cm). Phosphorus sensors were developed using a KaVo ScanXam^™^ scanner.

## Results

The dentition of the Altamura Neanderthal is 80% complete; all the teeth are present *in situ* except for the right P3 and left M1 in the maxilla and the right I1 and P3 and left I1 and I2 in the mandible. Observations were made on the following features: *ante-mortem* tooth loss, periodontal status, dental wear, palatine torus, taurodontism, retromolar space and periapical lesion of the upper I2.

### *Ante-mortem* tooth loss

**Fig 3:**
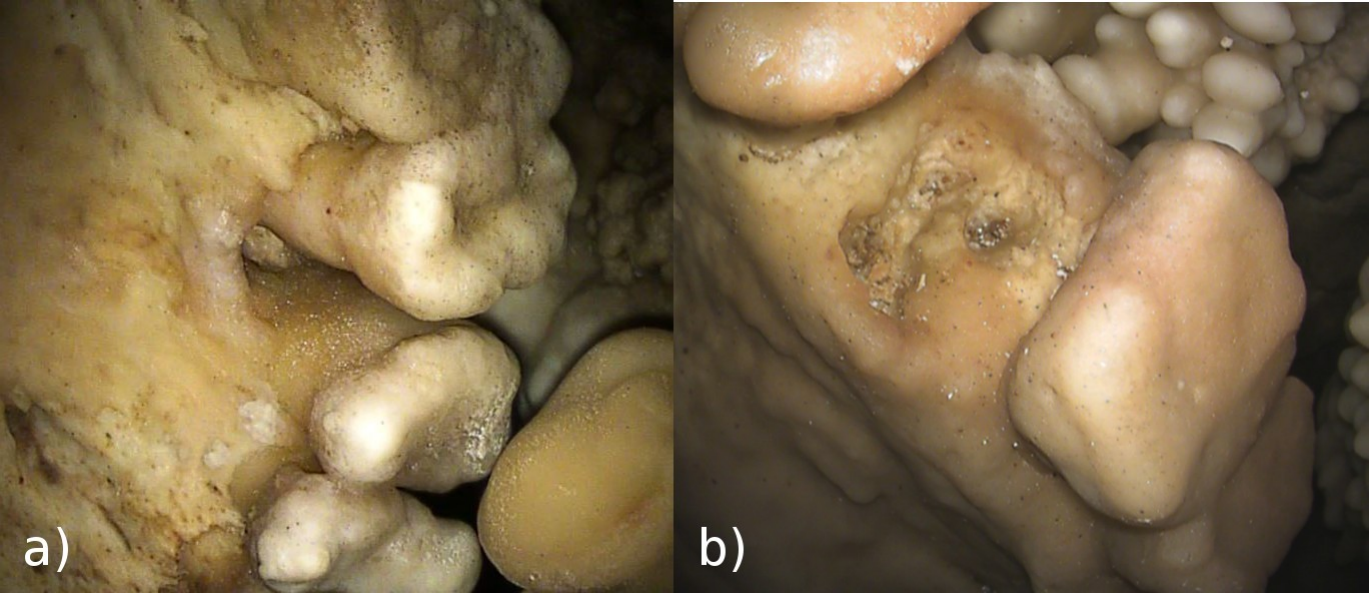
*Ante-Mortem* Tooth Loss (AMTL). a) Maxillary right hemiarch with (from above): M^1^, P^4^, C’, I^2^; between P^4^ and C’ there is a space with the remnants of the socket of RP^3^; the space left in the tooth row by the tooth loss is reduced by the relative drift of adjacent teeth. b) Socket of LM^1^ with a well-defined space for three roots; there are no evident marks of bone resorption. We cannot exclude the possibility of a *post-mortem* tooth loss, but the position of the skull makes this hypothesis unlikely.

The position of the cranium allows us to assume that the two missing maxillary teeth (RP^3^ and LM^1^) were lost *in vitam*. In the place for the RP^3^ (Fig 3A), the remnants of the alveolus are not resorbed and a deep gap is evident in the alveolar bone; the space left in the tooth row by the tooth loss is reduced due to migration of the adjacent P4.

The socket of LM^1^ is evident, although a calcareous film over the bone makes its observation difficult. The socket shows a well-defined trifurcation of the space for the roots (Fig 3B).

In the mandibular dentition, the calcite deposits prevent observation of the alveolar bone and make it difficult to reconstruct whether tooth loss of the anterior teeth occurred *in vitam* (as a consequence of alveolar resorption) or *post mortem*.

AMTL has been observed in a few Neanderthal specimens (Table 1). In one case (Gibraltar 2), the specimen is a child about 5 years old [9]; all the other specimens are advanced in age. Literature reports are not in agreement on the exact number of AMTL [10, 11]; however, in 2 of the 5 specimens (Guattari 1 and La Chapelle-aux-Saints 1), AMTL occurred in at least 25% of the observable alveoli.

**Table 1:** Occurrence of AMTL in Neanderthals. In the AMTL column the first number is the minimum (of only definitive cases) and the second number is the maximum (including probable cases).

Site	Specimen	Fossil	AMTL (min-max)	n alveoli	References
Gibraltar Forbes’ Quarry	Gibraltar 1	Cranium	0–3	16	[10, 11]
Gibraltar Devil’s tower	Gibraltar 2	Cranium + Mandible	1	19	[10, 11]
Grotta Guattari	Guattari 1	Cranium	7–16	28	[10, 11]
Grotta Guattari	Guattari 2	Mandible	1–3	12	[10, 11]
La Chapelle-aux-Saints	La Chapelle-aux-Saints 1	Cranium + Mandible	7–16	28	[10, 11]

### Periodontal status

Periodontium, the set of tissues that support the tooth in the jaw, is composed of alveolar bone, periodontal ligament, gingiva and cementum [12]. Pathological inflammation of the periodontium results in periodontal disease, a condition that may affect soft tissues (e.g. gingivitis) or also the alveolar bone, causing its resorption [13]. In archaeological and paleo-anthropological materials, periodontal disease is detectable only when it affected alveolar bone.

In the Altamura specimen, the marks of periodontal disease are present in correspondence of the lost RP^3^. Alveolar resorption is advanced and a deep lesion with remodeling of the alveolar margin is still present (Fig 3A). In correspondence of the other lost tooth (LM^1^), there is an incipient resorption of the buccal alveolar margin (Fig 3B). In both cases, the periodontal condition is localized and linked to the events that led to loss of the teeth.

**Fig 4:**
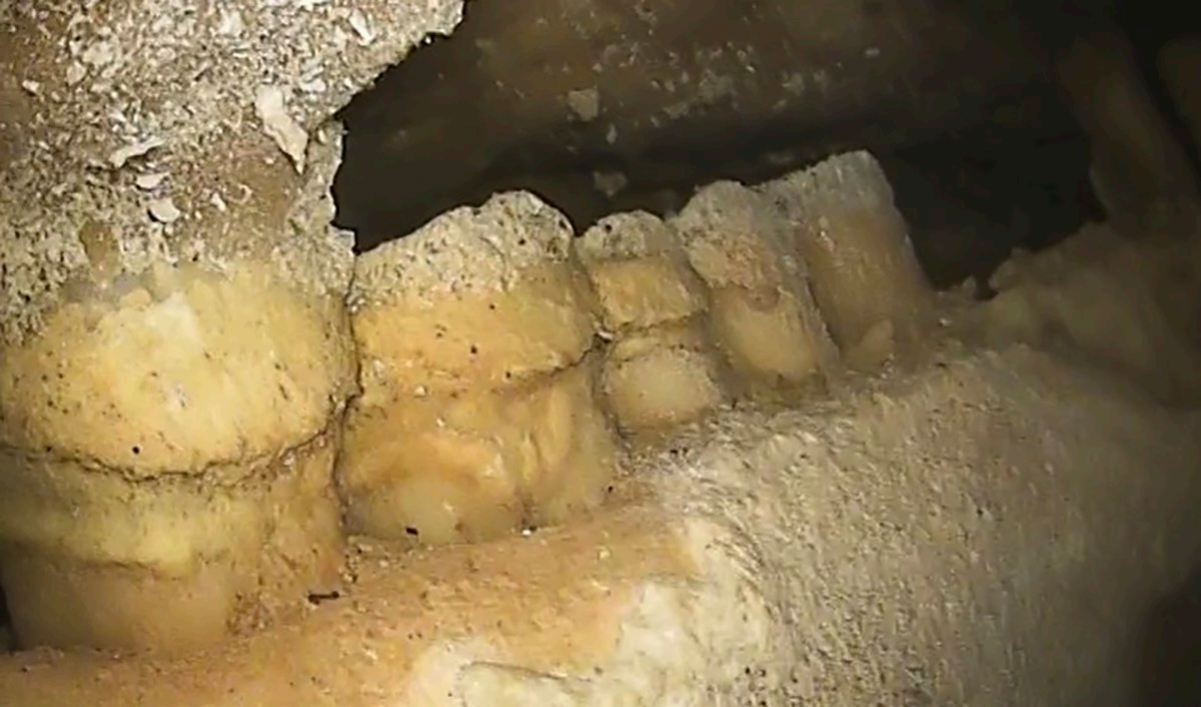
Root exposure on lower left molars. Left ramus of the mandible with (from the left): M_2_, M_1_, P_4_, P_3_, C,. The calcite layer covering teeth and bone prevents observation of the morphology of the alveolar bone. Despite the absence of clear marks of periodontal disease, there is evidence of root exposure of several millimeters.

On the remaining portions of the dental arcades, the calcite layer prevents observation of the morphological markers of periodontal disease visible on the bone, such as foramina, grooves, depressions or pockets. However, the general appearance of the alveolar margins does not appear to be consistent with severe periodontal disease. Nevertheless, there is evidence of marked root exposure, with a distance between the alveolar margin and the cemento-enamel junction (CEJ) sometimes of several millimeters (Fig 4). In the anthropological literature, root exposure is linked to two different mechanisms: alveolar bone resorption (due to periodontal disease) and compensative eruption [14, 15]. Several methods have been developed to distinguish between the causes [15–17]; these methods base the diagnosis of periodontal disease on the texture of the alveolar bone, assuming the healthy bone to be smooth and with no (or a few) interruptions by foramina, depressions and grooves. Until the calcite layer covering the bone can be virtually removed, it will be impossible to determine the state of the alveolar margin and the reason for the large extent of root exposure.

**Fig 5:**
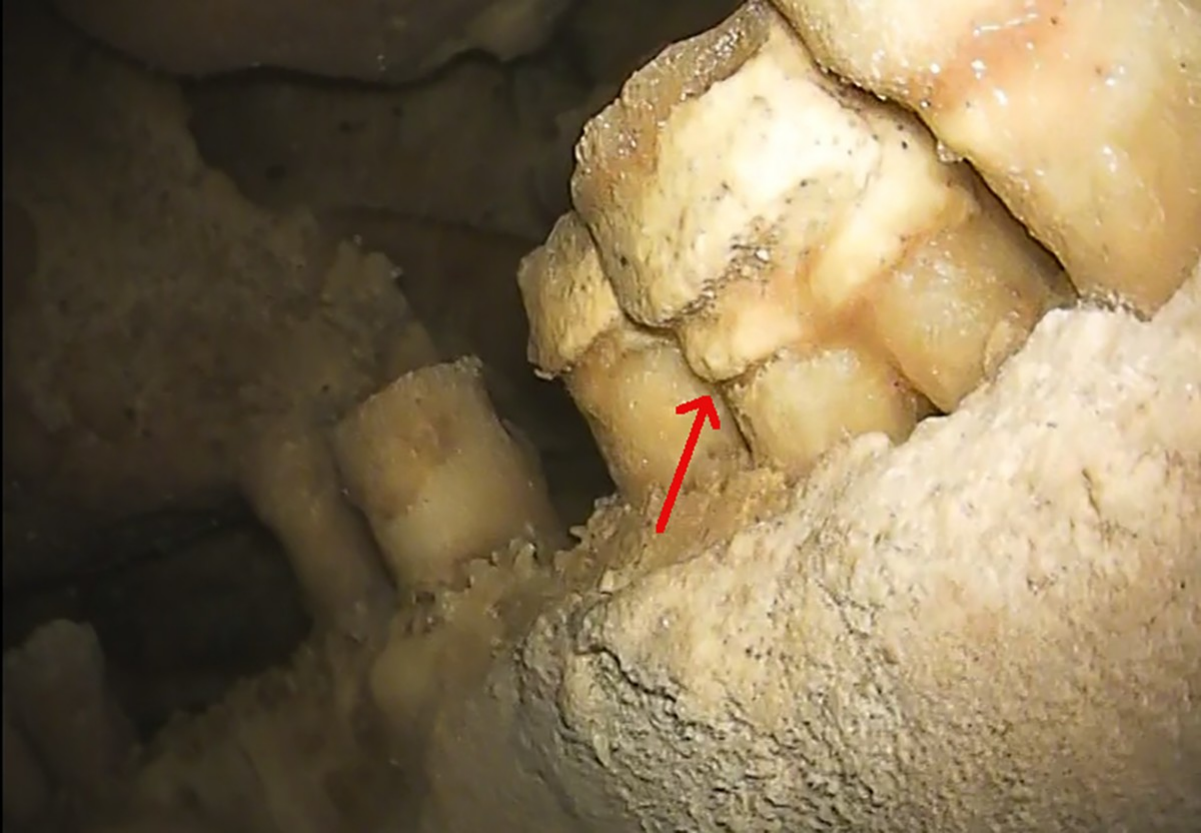
Dental calculus and taurodontism. On the lower right molars, as well on other teeth, dental calculus is present below the CEJ (red arrow). The position of the root furcation in RM_1_ is at about half of the tooth height and can thus be classified as hypotaurodont, following [20].

In some teeth, especially in the mandible, deposits of dental calculus are present under the calcite layer (Fig 5). Dental calculus is mineralized bacterial plaque and occurs on the crown or the root surface [12, 18]; in the living, it may irritate the gums and, together with dental plaque, is among the main causes of gingivitis and periodontal disease [19]. In this specimen, observations suggest that calculus deposits are just below the CEJ, above the edge of the alveolar bone and above the root furcation. This condition suggests that the periodontal tissue near the bone was healthy, with no evidence of any active periodontal disease.

### Dental wear

It is well established that observations on dental wear of ancient populations can provide insights into various aspects of their lifestyle. First, dental wear is age-related and, in each population, can contribute to the identification of individuals of older age (e.g. [21]). Second, dental wear depends on the diet of the individuals, and wear patterns can help to distinguish populations characterized by different diets [22]. Lastly, non-masticatory dental wear can provide information about cultural practices of past populations [23–25]. In the Altamura Neanderthal, observations on dental wear of the anterior teeth are impossible at the moment due to the thick coralloid concretions that totally mask the tooth surfaces (Fig 2). However, it is possible to make some observations on the posterior teeth, where occlusal wear is evident in the upper molars. The exact extent of the dental wear is difficult to quantify by traditional methods (e.g. [26]), although it is possible to briefly comment on dental compensating curves in the maxillary dentition only (the occlusal surfaces of the mandibular teeth cannot be observed). Erupted third molars indicate that the specimen was an adult, but further observation on dental compensating curves might provide a more precise age estimate.

**Fig 6:**
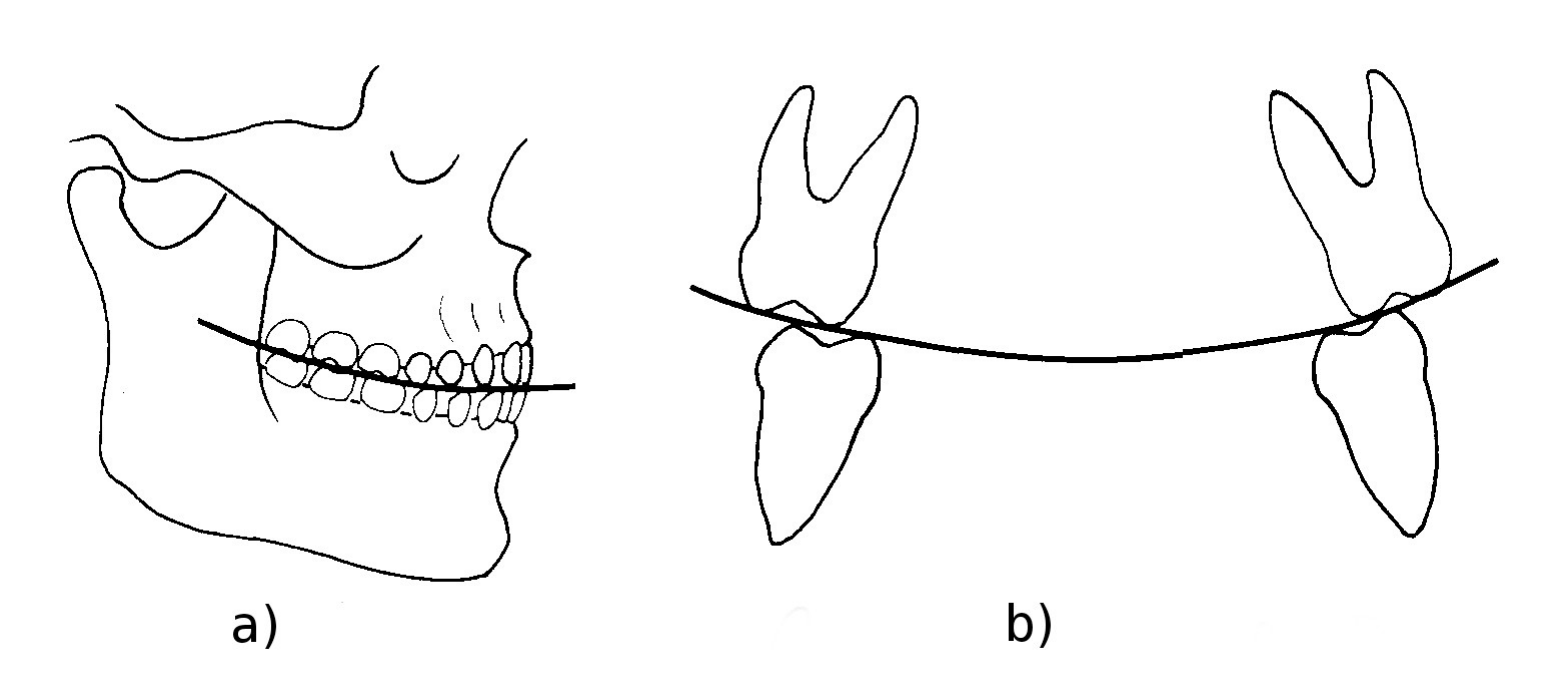
Dental compensating curves. a) The curve of Spee joins the cusp tips of the posterior teeth in lateral view. b) The curve of Wilson joins the cusp tips of each molar pair in frontal view (modified from [33]). Since the slope of the curves change as dental wear advances, observation of their status can be useful to assign an age range for the fossil.

**Fig 7:**
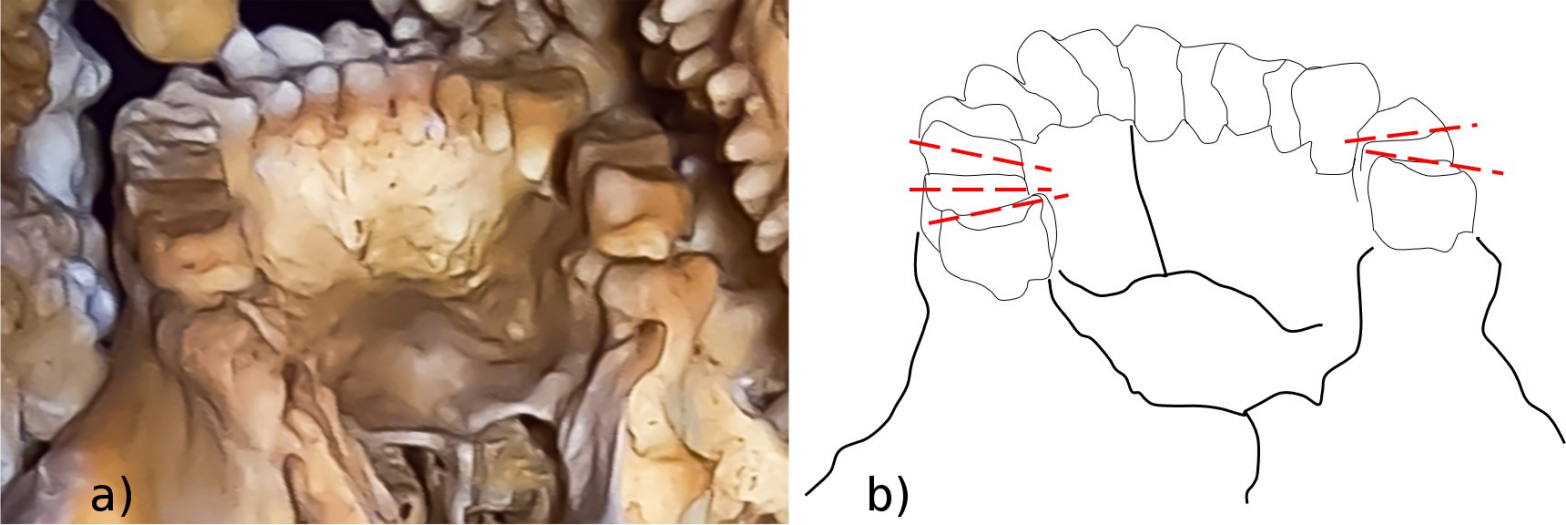
Curve of Wilson in the Altamura Neanderthal. a) Edited picture of the maxillary dental arcade from a posterior view. b) The tilting of the occlusal planes reveals an inverted curve of Wilson in the M^1^s and M^2^s and a normal curve in the M^3^s. This suggests that the individual was adult, but not elderly.

Dental compensating curves are curves along which the planes of the occlusal surfaces of the upper and lower jaw teeth lie. Two main curves are recognized [27–31]: in lateral view, the cusps of the posterior teeth lie on the curve of Spee (Fig 6A); in frontal view, the transverse curve joining the bucco-lingual posterior cusps of each pair of molars is called the curve of Wilson (Fig 6B). In unworn teeth, the curve of Wilson is concave on the mandible and convex on the maxilla, whereas in very worn molars the concavity of the curve reverses [32, 33]; this inversion is probably due to differential wear between the buccal and lingual cusps [34]. Although variation exists among individuals, populations and lifestyles [27, 35], the inversion is proportional to dental wear stage [27]; therefore, the inversion of the curve of Wilson occurs with different timing along the tooth row (M1 → M2 → M3). In modern human populations with an abrasive diet, inversion of the curve of Wilson can develop quickly after 20 years of age [33]. In the Altamura Neanderthal specimen, the status of the curve of Wilson can be observed in the maxilla, where an inverted curve is visible in the M^1^s and M^2^s, but not in the M^3^s (Fig 7). This suggests that the individual was fully adult, but not old, at the time of his death.

### Palatine torus

**Fig 8:**
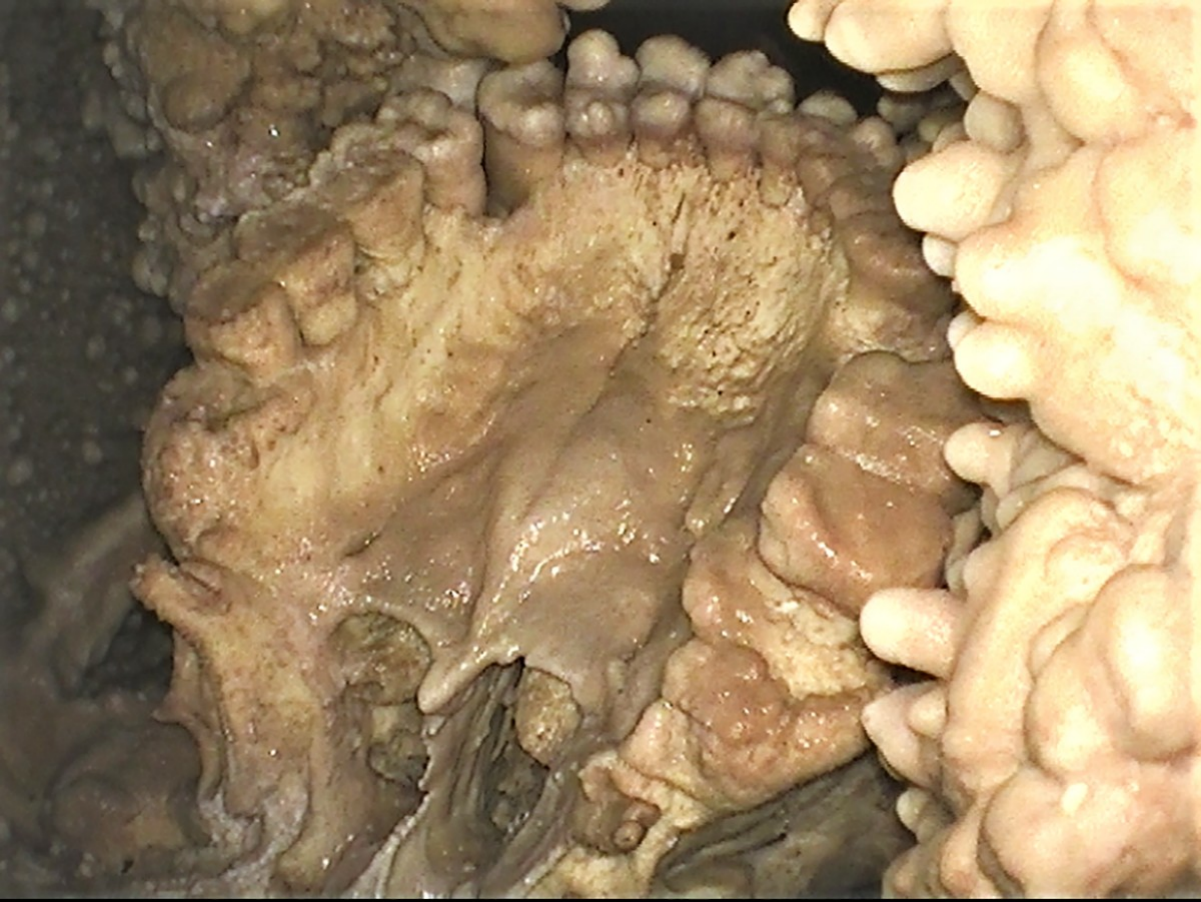
Palatine torus. View of the maxillary dental arch and palate. The well-marked bony exostosis on the median palatine suture of the Altamura Neanderthal can be scored as a grade 3 torus [36]. This is the first well-documented case of palatine torus in Neanderthals.

Palatine torus is defined as a bony exostosis occurring on the median palatine suture [35, 36]. In the Altamura Neanderthal, this feature is well marked (Fig 8) and can be scored at least as a grade 3 torus, following [36]. Table 2 lists the Paleolithic *Homo* specimens where the palatine torus has been observed [9, 35, 37–39]. Most of them are assigned to *Homo sapiens*; however, a torus has also been reported in the partial cranium D2282 from Dmanisi [9] and in the Middle Pleistocene hominin skull Kabwe 1 [39]. Among Neanderthals, palatine torus has been reported only for Guattari 1, the Neanderthal skull from Monte Circeo [35, 38] (but see [40]).

**Table 2:** Occurrence of palatine torus in the genus *Homo*. Fossil specimens of the genus *Homo* in which a palatine torus has been described.

Site	Specimen	Fossil	Palatine torus	References
Abri Pataud	Pataud 1	Cranium	Low, narrow, triangular torus	[37] p. 36
Chancelade	Chancelade 1	Almost complete skeleton	Low but expansive	[9, 37] p. 91
Cro-Magnon	CRM 1	Cranium and postcranial elements	Low maxillary torus	[9] p. 104
Dmanisi	D2282	Partial cranium	Low but distinct posteriorly	[9] p. 118
Grotta Guattari	Guattari 1	Cranium	Very irregular palatal surface	[38, 40]
Isturitz	Ist II #72 and #73	Maxillae	May bear maxillary torus	[9] p. 200
Jebel Irhoud	Jebel Irhoud 1	Cranium	Distinct palatal torus posteriorly	[39] p. 99
Kabwe	Kabwe 1	Cranium	Divided, with central sulcus	[39] p. 110
Mladec	Mladec 1	Cranium	Possible slight maxillary torus	[9] p. 274
Mladec	Mladec #5487	Maxillary fragments	Possible low torus	[9] p. 275

However, in some of the listed cases (Isturitz, Mladec 1, Mladec palate #5487), the authors are not sure of the presence of the torus, whereas in some others (Kabwe 1, Guattari 1) they may refer to something different from palatine torus *sensu stricto* as defined in [36]. Indeed, in the Kabwe 1 skull, the torus is described as “divided, with central sulcus” ([39], p.110), although Scott and Irish [36] exclude this kind of bony exostosis from the definition of palatine torus. In the case of Guattari 1, the situation is not straightforward. In their analysis of cranial epigenetic traits in the Neanderthal sample, Manzi et al. [40] comment that “The superficial osseous layer of the hard palate is destroyed and the area involved does not permit the usual scoring of a palatine torus; however, according to Sergi et al. (1934), the spongy substance visible along the midline could indicate the presence of a well developed palatine torus.” Following his original observations, Sergi himself described a very irregular palatal surface characterized by “many knobby and transversally oriented, elongated protrusions” ([38], p. 189). Direct observation of the Guattari 1 cranium by one of the present authors (AR) suggests the absence of a palatine torus as defined in [36].

### Taurodontism

Taurodontism, i.e. enlargement of the pulp chamber with a concomitant apical shift of the root furcation, is a feature also described in *Homo sapiens* but usually occurring with high frequency in the posterior dentition of Neanderthals [41–44]. Some authors have suggested that this trait could be an adaptation to high masticatory loads [43], but a more recent study argued that the relationship between masticatory biomechanics and an enlarged pulp chamber is weak and thus taurodontism is likely the result of genetic drift or other non-adaptive evolutionary mechanisms [44]. In the Altamura specimen, exposure of the molar roots allows some observations on this trait. In RM_1_, the position of the root furcation can be estimated at about half of the total tooth height (Fig 5); this condition can thus be classified as hypotaurodont, following [20]. In the other molars, despite the marked exposure of the roots, no root furcation is evident; in the maxillary molars, this could be linked to the thick calcite layer covering the roots and thus preventing any observation, whereas in the mandibular molars, where the calcite layer is thinner and the root surface visible, it probably suggests that they are characterized by weak taurodontism as well.

### Retromolar space

The retromolar space is a separation between the mandibular third molar and the anterior margin of the mandibular ramus [45]. Although it can be present in modern humans (e.g. [46]), it is recognized as a typical Neanderthal feature [47]. Most explanations proposed for the presence of the retromolar space in Neanderthals are based on their particular facial and/or mandibular morphology [48–52]; sometimes, anterior dental loading is mentioned as a supplementary explanation [53], although this idea was recently disputed [54].

**Fig 9:**
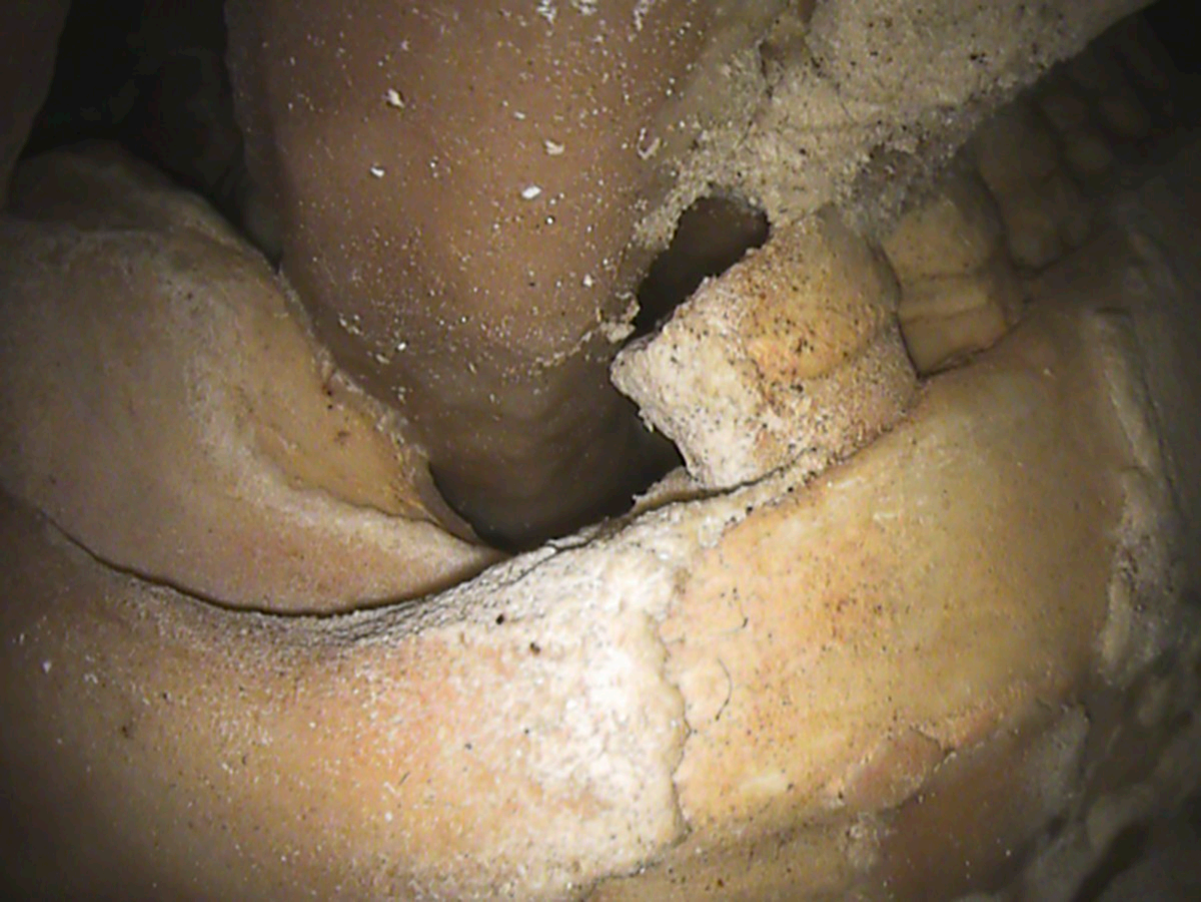
Retromolar space. In the posterior part of the mandible (right side shown in the picture), there is a wide space distal to M_3_, a trait characteristic of the Neanderthal lineage.

The retromolar space is present in the Altamura mandible (Fig 9). This is consistent with the variability of European Middle Pleistocene hominins, as it is a frequent trait in both Neanderthals and other European Middle Pleistocene hominins linked to the Neanderthal lineage, such as the Sima de los Huesos hominins [55]. The retromolar space can thus be added to the list of Neanderthal features already described for the Altamura specimen [3, 6, 8].

### Periapical lesion of the right upper I2

**Fig 10:**
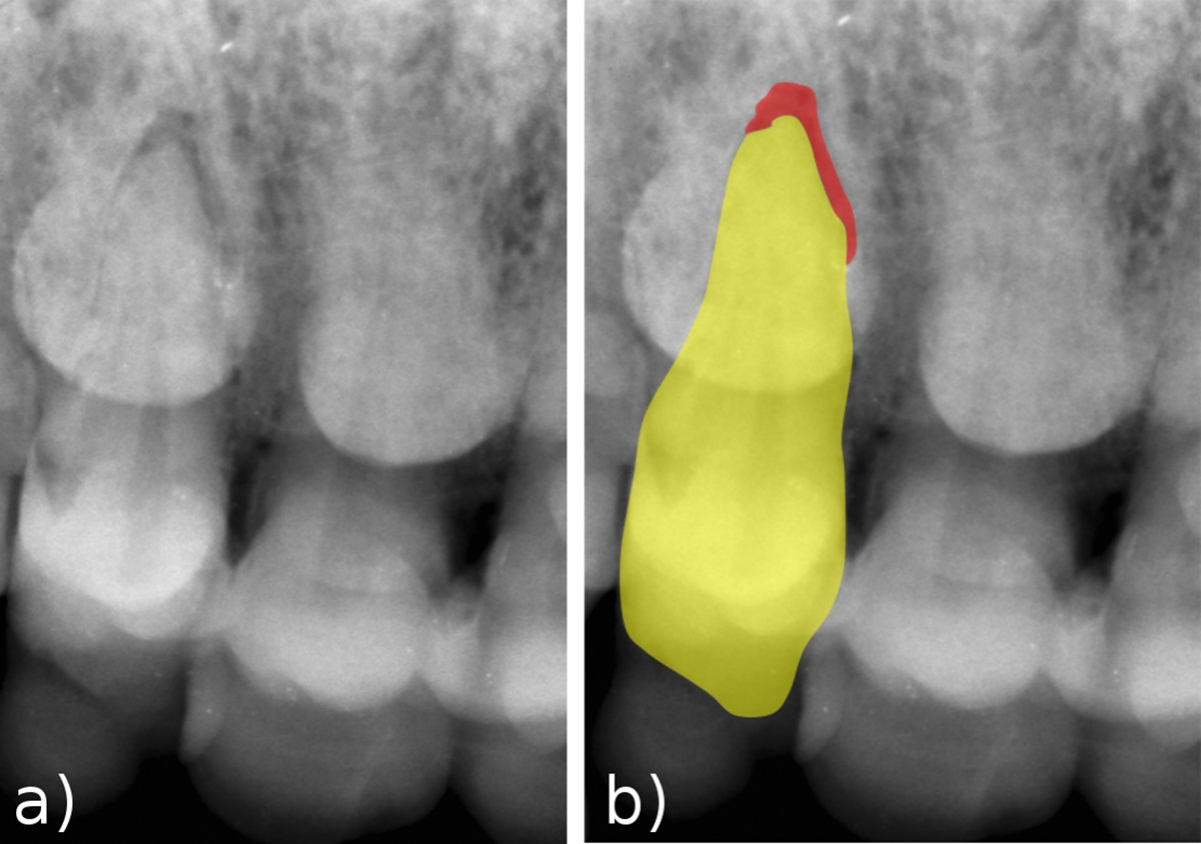
Periapical lesion of RI^2^. a) X-ray image of the upper right incisors, showing a void at the apex of the right I^2^ root. b) The superimposed graphic helps in identifying the outline of the tooth (yellow) and the periapical void (red), here interpreted as a lesion.

The position of the skull, embedded in coralloid formation, and of the mandible surrounded by other bones (Figs 1 and 2) did not allow easy positioning of the X-ray generating device and the receptor. For this reason, we were able to record only a single radiographic image at the level of the maxillary anterior teeth (Fig 10). The image shows an evident void between the tip of the root of RI^2^ and its alveolus, which we interpret as a periapical lesion.

Periapical lesions, sometimes associated with abscesses, have been reported for several Neanderthals (Table 3) [10, 56–60]. The prevalence of periapical lesions is similar for Neanderthals and Middle Paleolithic modern humans and slightly increase in Early Upper Paleolithic specimens [10]. Periapical lesions not resulting in an abscess are only identifiable using X-rays. Therefore, the number of periapical lesions in fossils and in the Altamura Neanderthal may be underestimated.

**Table 3:** Occurrence of periapical lesions in Neanderthals. All the lesions reported here resulted in an abscess, except that from Palomas identified by means of X-rays [59].

Site	Specimen	Teeth with periapical lesions	References
Amud	Amud 1	I^2^	[10]
El Sidrón	SDR 7–8	M_3_, M_1_, C, I_2_	[10, 58, 60]
Gibraltar Forbes’ Quarry	Gibraltar 1	P^4^, C’	[10]
La Chapelle-aux-Saints	La Chapelle-aux-Saints 1	C’	[10, 57]
La Ferrassie	La Ferrassie 1	P_4_, P_3_, C, I_2_	[10, 56]
Palomas	Palomas 59	M_2_	[59]
Saccopastore	Saccopastore 2	P^3^	[10]
Vindija	11.41	M_1_	[10]
Vindija	11.45	M_1_	[10]
Zafarraya	mandible	M_3_	[10]

## Discussion

Despite the numerous problems in carrying out observations on the Neanderthal specimen from Altamura, such as the position of the skeleton in the cave and the calcite layers of variable thickness covering the surface of the teeth and bones, it was possible to record several interesting features. Some of them confirm previous hypotheses on the systematic position of the specimen whereas others provide insights into aspects of the Altamura specimen never observed before, such as health status and age at death.

The presence of taurodontism and a retromolar space, traits very frequent in Neanderthals and other European Middle Pleistocene hominins [43, 55, 61], is consistent with previous observations placing the specimen in the range of variability of the Neanderthal lineage [3, 6, 8]. A marked palatine torus was also documented. Palatine torus appears to be rare in the human fossil record and, to our knowledge, the Altamura Neanderthal is the first well-documented case of palatine torus in a Neanderthal. In modern humans, palatine torus is most common in East Asians and Native American populations, followed by Europeans, Africans and Pacific populations [36], but it is unclear if its presence is a good marker of ancestry. Most likely, the etiology of palatine torus is multifactorial, depending on ancestry, sex, age, diet, masticatory loads, climate and environmental factors (for reviews see [35, 62]).

Dental wear and the inversion of the curve of Wilson in the first two molars suggest that the specimen is a fully adult individual, although not old. Therefore, the two cases of *ante-mortem* tooth loss (AMTL), i.e. RP^3^ and LM^1^ (6.25% per-alveolus), are not unexpected. This is consistent with some observations on AMTL prevalence in Neanderthals versus Middle and Early Upper Paleolithic modern humans, revealing a higher prevalence in the former than in the latter (4.9%-5.8% vs 0.5%-2.4% per-alveolus) [10]. It is reasonable to suggest that RP^3^ must have been lost at least several months before death, since the other teeth of the right maxillary arcade had enough time to rearrange their position along the tooth row, leading to a reduction of the space between C’ and P^4^. LM^1^ must have been lost a few weeks before death, since alveolar resorption is still in an incipient stage. In modern humans, complete resorption of the socket generally occurs in a few months [63, 64]. Since there are no signs of bone resorption, we must consider the possibility that LM^1^ was held in the socket by the soft tissues and was lost *post mortem*.

**Fig 11:**
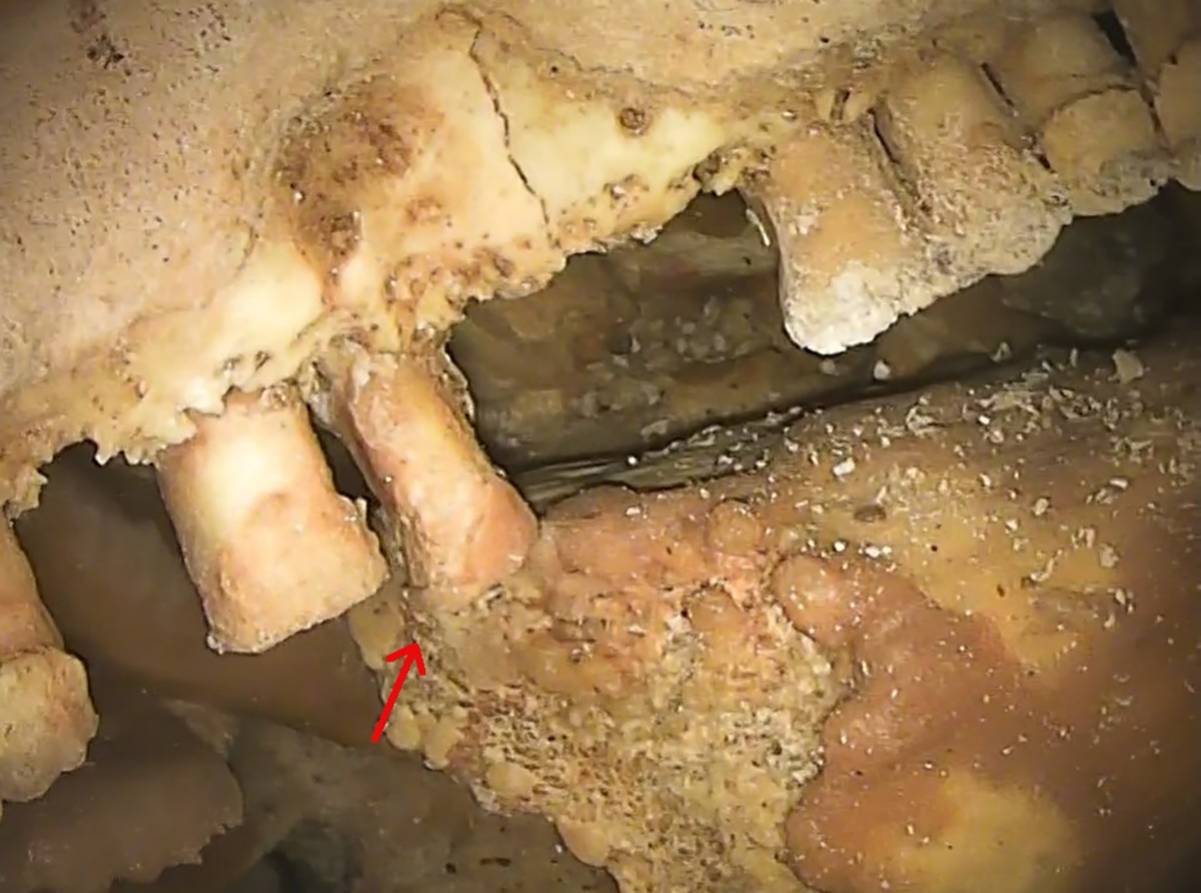
*Post-Mortem* Tooth Loss (PMTL) in the mandible. The upside-down position of the mandible may have facilitated PMTL of LI_1_, LI_2_, RI_1_ and RP_3_. The red arrow points to RI_2_, partially dislodged from its socket and sustained by a rib below the mandible.

It is reasonable to hypothesize that the loss of LI_1_, LI_2_, RI_1_ and RP_3_ occurred *post mortem*, since the position of the mandible in the site (with the dentition facing the cave floor) may have facilitated dislodgement of the single-rooted teeth from the socket. In this regard, it is notable that RI_2_, which is partly dislodged from the socket, was prevented from falling out completely by the presence of a rib below the mandible (Fig 11). In both cases, AMTL was probably linked to single, isolated traumatic or pathological events that did not affect the rest of the dentition, and thus was not a consequence of extreme alveolar bone resorption. Indeed, the rest of the alveolar bone appears to be in good condition, given the adult age of the specimen, and there are no visible marks of periodontal diseases, although dental calculus is present near the CEJ in the posterior dentition. At present, it is not possible to evaluate the exact occurrence and extent of periodontal diseases, their kind and severity; also, the possibility that the observed degree of root exposure was the result of compensatory eruption [14, 15] cannot be further explored.

The use of an X-ray device made it possible to observe a periapical lesion in RI^2^. Openings in the dental hard tissues (such as dental wear, traumas, caries, etc.) are among the most common routes of bacterial infection of the pulp chamber, and the infection can spread through the root canal system to the periapex [65]. In the Late Pleistocene, periapical lesions are rarely of carious origin [10]. This, together with the observation of marked wear at least on the molars, favors the hypothesis of a periapical lesion linked to extreme dental wear of the incisal margin.

To further clarify functional details of the Altamura Neanderthal dentition, future research will require micro-CT scanning of the entire maxilla and mandible.
